# A Case of Strangulated Urethral Prolapse in a Premenopausal Adult Female

**DOI:** 10.1155/2016/1802623

**Published:** 2016-06-20

**Authors:** Morris L. Jessop, Stanley Zaslau, Osama Al-Omar

**Affiliations:** Division of Urology, West Virginia University, P.O. Box 9251, Morgantown, WV 26506, USA

## Abstract

Urethral prolapse in a premenopausal adult female is exceedingly rare. This paper describes a case of strangulated urethral prolapse presenting as a urethral mass in an unusual demographic and reviews the literature on etiology and management. Only a few cases have occurred in women of reproductive age. The etiology is likely multifactorial. Treatment with surgical excision provides good results in the majority of cases.

## 1. Introduction

Urethral prolapse is defined as eversion of the urethral mucosa through the external urethral meatus [1]. The vast majority of cases occur in the pediatric population (80%), with the remaining cases occurring primarily in white postmenopausal females [2]. The incidence in the pediatric age group has been estimated to be approximately 1 : 3000. Interestingly, over 90% of urethral prolapse in the pediatric age group occurs in females of African descent [3]. Patients in the pediatric population are usually asymptomatic on presentation, and often urethral prolapse is an incidental exam finding [4].

In contrast to the pediatric population, urethral prolapse in adults is extremely rare and occurs almost exclusively in white and postmenopausal females. Vaginal bleeding, hematuria, dysuria, frequency, urgency, and nocturia are all potential presenting symptoms [5]. Herein, we present the case of urethral prolapse in a woman of reproductive age.

## 2. Case Presentation

A 46-year-old female presented to the emergency room with a bulging mass from her urethra. The patient reported progressing groin discomfort over the previous week that she had attributed at first to the onset of menstruation. The patient then felt a “bump” while wiping and discovered with a mirror a large mass bulging from her urethra. She subsequently went to a rural emergency room for evaluation, where the treating physician placed a Foley catheter. She was subsequently transferred to our tertiary institution for urologic evaluation.

Subjectively the patient complained of some pain with palpation of the mass. She denied any urinary complaints, even prior to this event, including hematuria, dysuria, frequency, or difficulty voiding. She denied any trauma. Her medical history was remarkable for hypothyroidism and untreated but mild stress urinary incontinence that had been present since her last vaginal birth 14 years prior. The patient reported a total of 2 prior vaginal deliveries. Her past surgical history was remarkable for an endometrial ablation procedure and breast augmentation. She had also recently undergone laser hair removal of her genital pubic hair.

**Figure 1:**
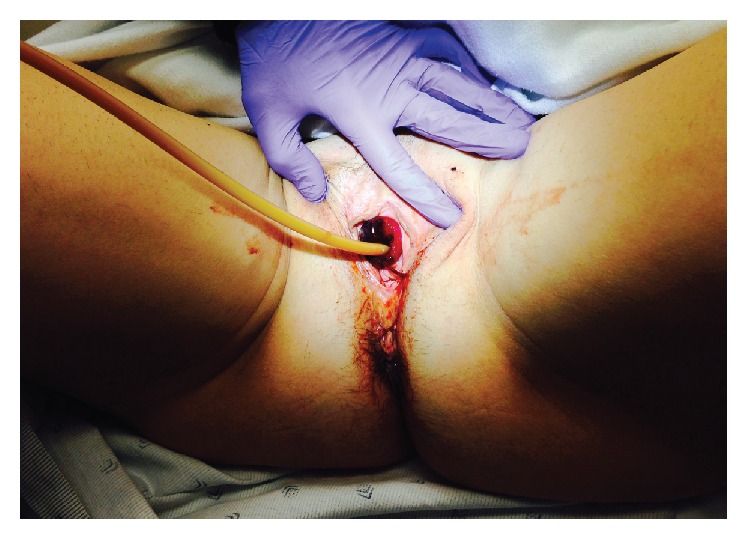
Appearance of strangulated urethral prolapse on physical exam.

Her vitals were within normal limits. On exam, the patient was a well-developed, nonobese female (BMI 22.3) in no acute distress. Her genitourinary exam was remarkable for prolapsed, congested, and necrotic urethral mucosa surrounding the Foley catheter (Figure 1). There was no palpable cystocele or rectocele. The remainder of her exam was unremarkable. Attempts at manual reduction of the urethral prolapse without general anesthesia were not tolerated.

**Figure 2:**
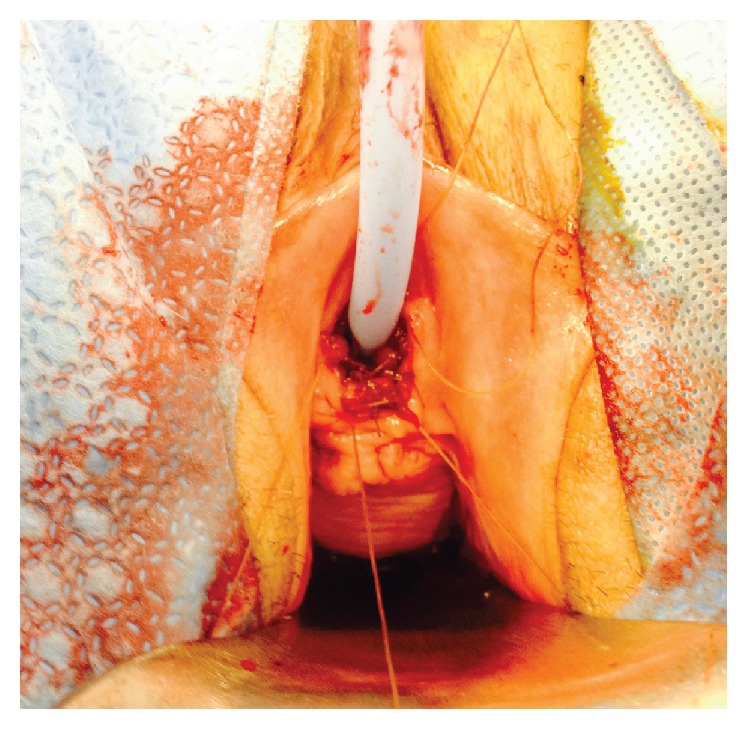
Postsurgical appearance with the prolapsed tissue excised from the healthy urethral margins sutured to the vestibule.

The patient was brought to the operating room. The catheter was removed. However, attempts at manual reduction when anesthesia was induced were not successful. Cystourethroscopy was performed revealing excess urethral mucosa. The bladder was otherwise unremarkable. A new catheter was placed. Using electrocautery, the prolapsed urethral mucosa was circumferentially excised around the catheter. At intervals, the healthy urethral mucosal margin was sutured to the vestibule using absorbable suture. The final result yielded all prolapsed tissue excised from healthy urethral margins that were sutured to the vestibule (Figure 2). At the end of the operation, a generous amount of estrogen cream was applied. The patient tolerated the procedure well. The excised tissue was sent for permanent pathology, which was later described as urethral mucosa with congested, thrombosed, and dilated vessels. The patient was discharged the following day with the catheter in place and instructions to remove the catheter in 7 days. She was also instructed to continue to apply estrogen cream daily until follow-up. At most recent follow-up, over 1 year since her procedure, she is voiding well and with no evidence of incontinence.

## 3. Discussion

The etiology of urethral prolapse is controversial and multiple theories have been proposed. One popular theory is that it occurs from weak attachments between the inner and outer smooth muscle layers of the urethra [3]. Other congenital and acquired causes have also been implicated, including genetics, abnormal urethral anatomy, underlying neuromuscular disorders, or deficiencies in elastic tissues. Known acquired risk factors include conditions that chronically increased intra-abdominal pressure including asthma, obesity, and constipation. Additionally, several cases of urethral prolapse have occurred after injection of bulking agents [6]. Another possible risk factor is estrogen deficiency, which commonly occurs after menopause [7]. The urethral mucosa and submucosal tissues are sensitive to estrogen and potentially lead to a mucosal seal. Estrogen deficiency could in turn lead to weak periurethral fascia and when superimposed on the risks factors previously described could potentially lead to urethral prolapse.

Diagnosis of urethral prolapse is made by history and clinical findings of a circumferentially prolapsed meatus, often described as a “doughnut” shaped mass protruding from the urethra. It may be confirmed by placement of a catheter or cystoscopy. The prolapsed urethra may be congested, necrotic, bleeding, and tender to palpation. The differential diagnosis includes malignancy, condylomas, and urethral caruncle, the latter can be differentiated by the lack of circumferential urethral involvement. Extensive imaging, lab work, and other testing for workup is almost always unnecessary.

For nonstrangulated prolapse, medical therapy consists of topical estrogens and good hygeine. Sitz baths may also be attempted with mostly good success reported in children [2]. Recurrence after conservative therapy or the presence of strangulation requires surgery. Several surgical techniques have been described, the most popular being excision of the prolapsed mucosa and reapproximation of the mucosal margins with absorbable suture. However, other methods have been performed including urethral plication, reduction of the prolapse and placement holding sutures (vesicourethropexy), and cautery excision [1].

To our knowledge, only four cases of urethral prolapse in adult premenopausal females have been described to date [1, 8, 9]. The last case was described in a 46XY phenotypic female with androgen insensitivity syndrome [10]. The ages of presentation, including our case, were variable at 18, 38, 39, 46, and 48. With the exception of the 48-year-old patient, symptoms at presentation included dysuria, frequency, and vaginal bleeding, the latter being described as the primary presenting symptom in all but one of the cases. All but one of the cases were treated with surgical excision. Results with surgery were reported as excellent in all cases.
